# The Synthesis of Hybrid Gold-Silicon Nano Particles in a Liquid

**DOI:** 10.1038/s41598-017-09634-y

**Published:** 2017-08-31

**Authors:** S. Kutrovskaya, S. Arakelian, A. Kucherik, A. Osipov, A. Evlyukhin, A. V. Kavokin

**Affiliations:** 1 Department of Physics and applied mathematics, Stoletov Vladimir State University, 600000 Gor’kii street, Vladimir, Russia; 2 Russian Quantum Center, 143025 Novaya street 100, Skolkovo, Moscow Region Russia; 3 Laser Zentrum Hannover e.V., Hollerithallee 8, 30419 Hannover, Germany; 4 ITMO University, 197101 Kronverksky Prospekt 49, St. Petersburg, Russia; 5 School of Physics and Astronomy, University of Southampton, Southampton, United Kingdom; 6 Spin Optics Laboratory, St. Petersburg State University, 198504 Ulyanovskaya street 1, St. Petersburg, Russia; 7 CNR-SPIN, Viale del Politecnico 1, I-00133 Rome, Italy

## Abstract

We show that the laser ablation method can be efficiently employed for the synthesis of silicon nanoparticles (NP), which are characterized by a strong resonant optical response in the visible spectral range. A single layer composed of silicon NPs has been deposited from the colloidal solution generated by laser ablation. The formation of hybrid silicon-gold NPs as a result of the laser action on a mixed colloidal solution is observed. These hybrid NPs are characterized by broadening of the near-field photoluminescence spectra compared to pure silicon NPs. These results may be used for the realization of functional metasurfaces consisting of randomly distributed resonant NPs.

## Introduction

Bulk silicon is an indirect gap semiconductor which is the rate of radiative recombination in silicon is orders of magnitude lower than in direct gap semiconductors, e.g. GaAs. The low radiative recombination rate limits applications of bulk silicon crystals in optoelectronic devices. Nanostructuring of silicon is a good tool for circumventing this obstacle. Silicon nanoparticles (NPs) show a highly efficient photoemission in the visible and near-infrared (IR) frequency ranges. The dramatic improvement of the radiative properties of silicon is a consequence of size quantisation of electrons and holes in nanoparticles that relaxes the wave-vector conservation rule and induces a much higher radiative recombination rates compared to the bulk crystal. Moreover, silicon NPs are characterised by remarkable photostability and allow for the wide-range tuning of the photoemission wavelength by altering the size of the nanoparticles. For these reasons, silicon NPs have a very large application area in the visible and near IR ranges^1, 2^. These applications are mostly based on the characteristic spectral response of silicon. The optical properties of NPs, including their spectral resonances, are essentially governed by their size, shape, and the crystallization degree^3^. The development of fabrication techniques that would enable a high-precision control over the NP parameters is very important in this context. The method of laser ablation in a liquid phase^3, 4^ offers a possibility to control the average size and shape of the synthesized particles by a proper choice of the irradiation conditions (pulse duration, energy density etc.). Low optical losses in silicon NPs are crucial for their applications in photonics, in particular as building blocks for metamaterials and metasurfaces. For these applications in is also important to be able to engineer the near field emission of silicon NPs. This work is aimed at the experimental demonstration of such tailoring and enhancement of the near field emission of silicon NPs by covering them by small-size golden NPs. Golden shells trigger a remarkable nanoantenna effect that strongly affects the near field emission of silicon.

Here we report on the synthesis of silicon NP by the continuous laser irradiation of a thin crystalline monolithic silicon target placed in ethanol. The possibility of formation of hybrid silicon-gold structures by laser ablation in colloidal solutions^5^ is demonstrated here. The increase of the optical near field magnitude in the emission of the NPs is observed and interpreted in terms of the redistribution of the near-field scattering intensity due to the nanoantenna action by gold nanoparticles. The spontaneous ordering of NPs in the course of their deposition allows for the formation of thin films that may be used for creation of metasurfaces suitable for controllable manipulation of the transmission and reflectivity of light^6, 7^. These results pave the way to applications of hybrid gold-silicon NPs in optical integrated circuits combining functions of generation, transmission and detection of optical signals.

## Results

### The synthesis of colloidal nanoparticles

In order to synthesize silicon NPs we have used the method of the CW-laser ablation^8, 9^. The application of a moderate intensity CW-laser radiation source enabled us to obtain NPs characterized by a small dispersion of the average size^4, 10^. In our experiments the targets of crystalline monolithic silicon were used. To avoid the oxidation of NPs we have conducted the laser ablation experiment in the 99% ethanol solution^3^.

The laser beam has been focused on the surface of the target using the lens with the focal length of 100 mm and the laser spot diameter of 100 μm. The power variation was in the range of 10–100 W, which corresponded to the radiation intensity of 10^5^–106 W/cm^2^. Estimating the time of laser irradiation by τ = *D/v* with *v* being the scanning speed, *D* being the laser beam diameter we conclude that the irradiation time varied in the range between 1 s and 10 s, corresponding to the scanning speeds in the range of 100 μm/s to 10 μm/s, respectively. The total irradiation time including all scans has been 60 minutes.

**Figure 1:**
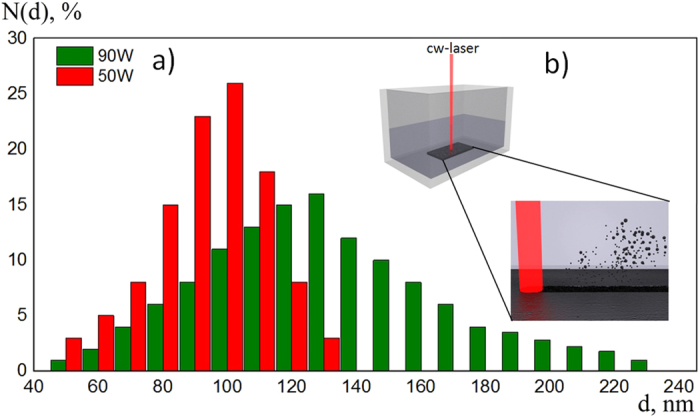
The histogram (**a**) showing the distribution of the particle diameters in a colloidal system realized by laser irradiation of the monolithic target. The laser power used was either 50 W (red bars) or 90 W (green bars), the scanning speed was 100 μm/s in average. The scheme of CW-laser ablation process of a silicon target is shown in the inset.

The increase of the laser irradiation power resulted in a wider distribution of NP sizes (Fig. 1, green bars). The mean diameter of spherical NPs was about 100 nm.

**Figure 2:**
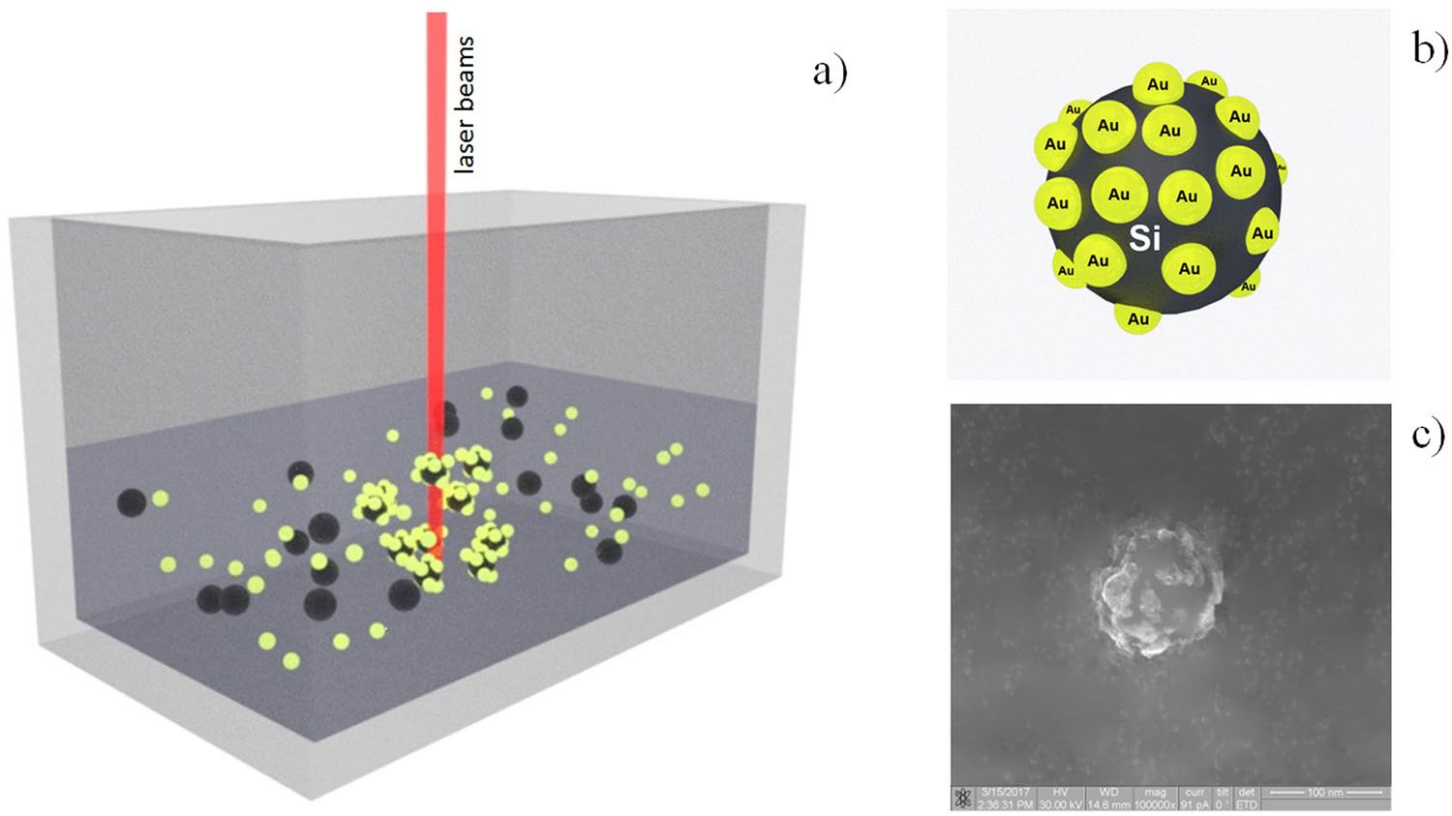
The schematic of the formation of hybrid NPs due to the laser irradiation of a mixed Si-Au colloidal solution (**a**). A schematic of the hybrid Si-Au particle (**b**). REM-image of a silicon colloidal particle covered by gold particles deposited from a colloidal solution (**c**).

Once the colloidal solution of silicon NPs was obtained we added gold particles of the 10 nm diameter into it. For the irradiation of mixed gold and silicon NP solutions, the Ytterbium fiber laser characterized by the wavelength of 1.06 µm, the pulse duration of 100 ns, the repetition rate of 20 kHz, and laser pulse energy up to 1 mJ was then used. The diameter of the laser beam at the focal plane was 30 µm. The laser irradiation of the mixed colloidal system resulted in the formation of hybrid gold-silicon NPs (Fig. 2).

The most likely mechanism of formation of the hybrid NPs is due to the electrostatic attraction of their constituents. The golden NPs are negatively charged as confirmed by the z-potential measurement^10^. The silicon particles are electrically neutral, initially. However, the non-radiative recombination of optically generated free carriers leads to the break of chemical bonds at the surface of silicon NPs, so that they acquire positive charges. As a consequence, positively charged silicon NPs attract negatively charged golden NPs that results in the formation of hybrid clusters (see Fig. 2b).

The hybrid nano-system we have obtained in this way allows for combining the properties of such prospective materials as gold and silicon^11^. The optical hybrid metasurfaces composed by golden and silicon NPs and emitting in the visible spectral range are expected to have a broad range of applications in opto-electronics and photonics.

### The results of the nps deposition

To control the deposition of the NPs from a small colloidal drop we taken advantage of the methods described in refs 8, 10 and 12 Namely, we have deposited silicon NPs using the technology of sputtering small colloidal drops on the surface of the transparent substrate.

**Figure 3:**
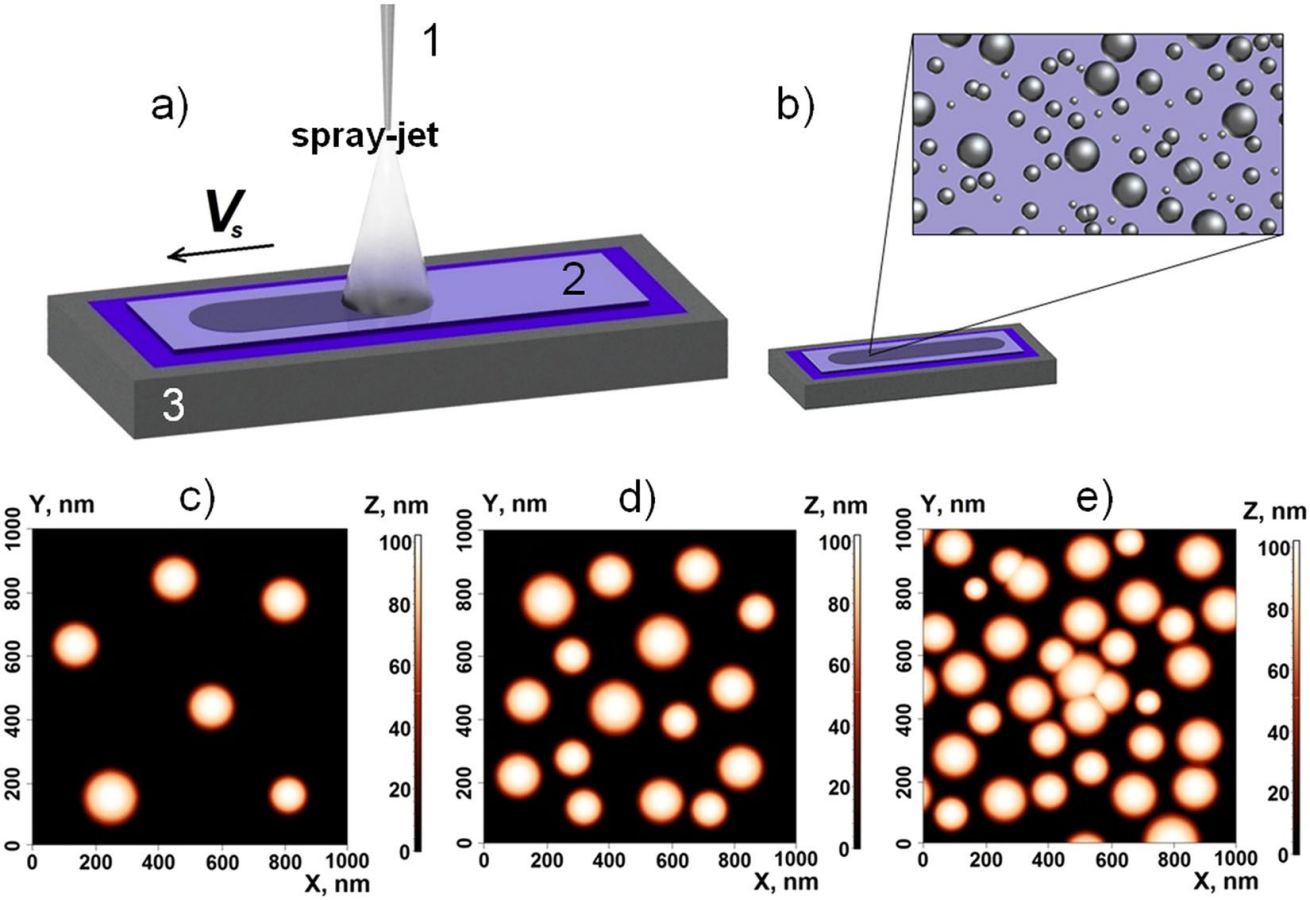
The colloidal particles deposition scheme (**a**): 1 is the capillary with a colloidal solution, 2 is the substrate, 3 is the thermostabilized one-coordinate table. The schematic showing the deposited particles (**b**). The AFM-images of the deposited silicon colloidal particles (**c**–**e**) on a stable temperature transparent substrate. The images correspond to the different scan velocities: 1 mm/s, 0.5 mm/s, 0.1 mm/s, respectively (left to right).

Colloidal systems have been sprayed through a capillary with a diameter of 100 μm (see Fig. 3). The system was pressurized by the pneumatic pump, and the pressure was about 5 bar. The capillary was positioned perpendicularly to the surface of the substrate at a distance of 100 mm. The glass substrate was fixed on the surface of a thermostabilized one-coordinate table with a temperature of 20 °C. The speed of the table movement (*V*_*s*_) was ranging from 0.1 mm/s to 1 mm/s. The deposited droplets took a spherical shape, that is what one would expect having in mind that the bond condition is satisfied in our system:

1 $${B}_{o}\equiv \frac{g(\rho -{\rho }_{m}){d}^{2}}{\sigma }\ll 1,$$

where *g* is the gravity acceleration index, *ρ* is the density of the droplet, ρ_m_ is the density of the environment, σ is the surface tension, *d* is the droplet diameter.

We have estimated the droplet evaporation rate may using the Maxwell diffusion model. The variation of the droplet height in time has been described by the following equation:

2 $$h(r,t)=\sqrt{{(\frac{h{(0,t)}^{2}+{R}^{2}}{2h(0,t)})}^{2}-{r}^{2}}-\frac{{R}^{2}-h{(0,t)}^{2}}{2h(0,t)},$$

where *h*(0, *t*) is the height of the droplet measured in its center, *R* is the droplet base radius.

We assume that in the linear approximation the height of the droplet varies as:^13^

3 $$h(0,t)={h}_{0}-{v}_{0}t,$$

where *v*_0_ is the speed of the height variation, *h*_0_ is the initial height (for a spherical drop *h*_0_ = R).

After depositing the ethanol drop onto the substrate, the molecules of liquid tend to move towards the solid surface^14^. The evaporation goes faster at the rim of the drop^15^. If NPs are suspended in an ethanol, the effective thermal conductivity dramatically increases in comparison to the pure liquid^16^ that means that the parameter ${v}_{0}$ increases for colloidal droplets. We estimate the evaporation time *t* (for ${{\rm{v}}}_{0}$ ~1.6 μm/s) to be ranging from 1.5 to 12 sec as a function of the droplet radius R that varies from 1 μm to 20 μm. This estimation agrees quite well with the experimental data.

The deposited layer structures obtained with different speeds of the surface scanning are shown in Fig. 3.

The decrease of the scanning speed results in the merging of small colloidal droplets, that is followed by the formation of agglomerates. In these experiments, the height of the deposited layer did not exceed 100 nm, which roughly corresponds to the diameter of the deposited particles.

### Optical properties of hybridparticles: a dark-field microscopy study

**Figure 4:**
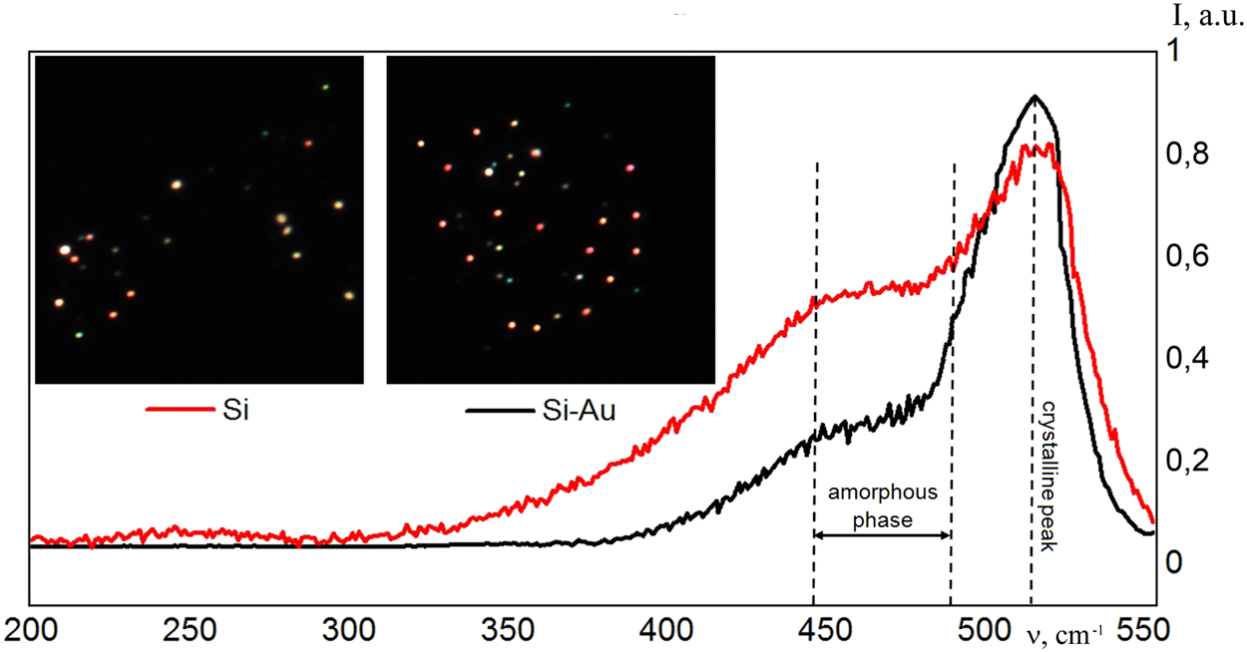
A dark-field (scattered light) image of the deposited Si NPs (**a**) and hybrid gold-silicon NPs (**b**). The observation area is 10 × 10 μm. The Raman spectra of Si NPs and of the laser irradiated Si-Au colloidal solution (**c**).

The conclusions of the above section on the geometry of the deposited NPs are confirmed by the Raman spectroscopy data (Fig. 4).

The Raman spectra taken from the initial colloidal system (the red curve in Fig. 4) show a peak at the wavelength of 520 cm^−1^ that corresponds to the crystalline phase of silicon and a broad shoulder (440–480 cm^−1^) corresponding to the amorphous phase of silicon. After the laser irradiation of the mixed Si-Au colloidal solution, the intensity of the peak corresponding to the crystalline phase (black curve in Fig. 4) is increased, and the shoulder corresponding to the amorphous phase is reduced.

**Figure 5:**
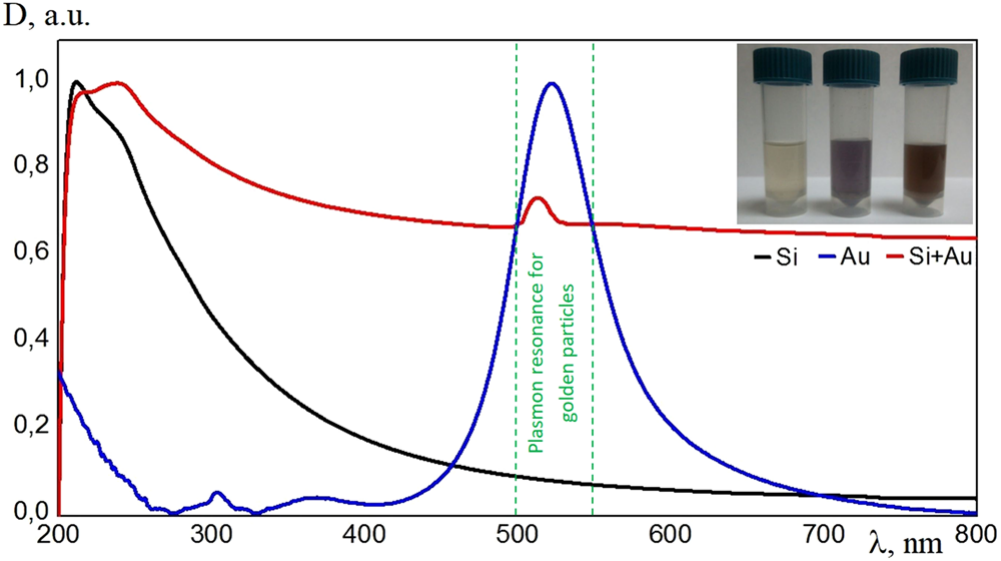
The optical density of colloidal solutions which contain Si NPs (black curve), golden NPs (blue curve) and hybrid gold-silicon NPs (red curve).

The optical spectroscopy studies of the synthesized colloidal systems demonstrate a significant modification of the light absorption spectra as a result of the association of silicon and gold nanoparticles. The initial colloid containing only silicon particles is characterised by the peak of absorption at the wavelength of about 200 nm followed by its exponential decay at the longer wavelength (black curve in Fig. 5). The absorption spectra of golden nanoparticles (blue curve in Fig. 5) demonstrate a broad plasmonic peak in the range of 500–560 nm. Now, the hybrid gold-silicon nanoparticles are characterised by a much narrower and lower amplitude peak at about 500–520 nm on the background of the exponential tail. This spectrum is indicative of a strong variation of the near field response of the hybrid nanoparticles compared to silicon nanoparticles in the range of 500–520 nm.

In order to have a deeper insight to the optical properties of the synthesized hybrid NPs we have used the dark field microscope^13^, where only the scattered light is collected. As one can see from Fig. 4a,b, NPs fabricated from the crystalline Si targets with addition of gold particles resonantly scatter light in a wide spectral range: from red to blue color. It is important to note that a large part of Si NPs does not provide any resonant optical response in the visible range, because of their low refractive index. After the laser irradiation that led to the merging of Si and gold NPs and formation of clusters we detected a much stronger scattering signal from the deposited NPs.

To reveal the optical properties of the synthesized hybrid NPs we have compared the images provided by the atomic force microscope (AFM) with those of the scanning near-field optical microscope (SNOM).

**Figure 6:**
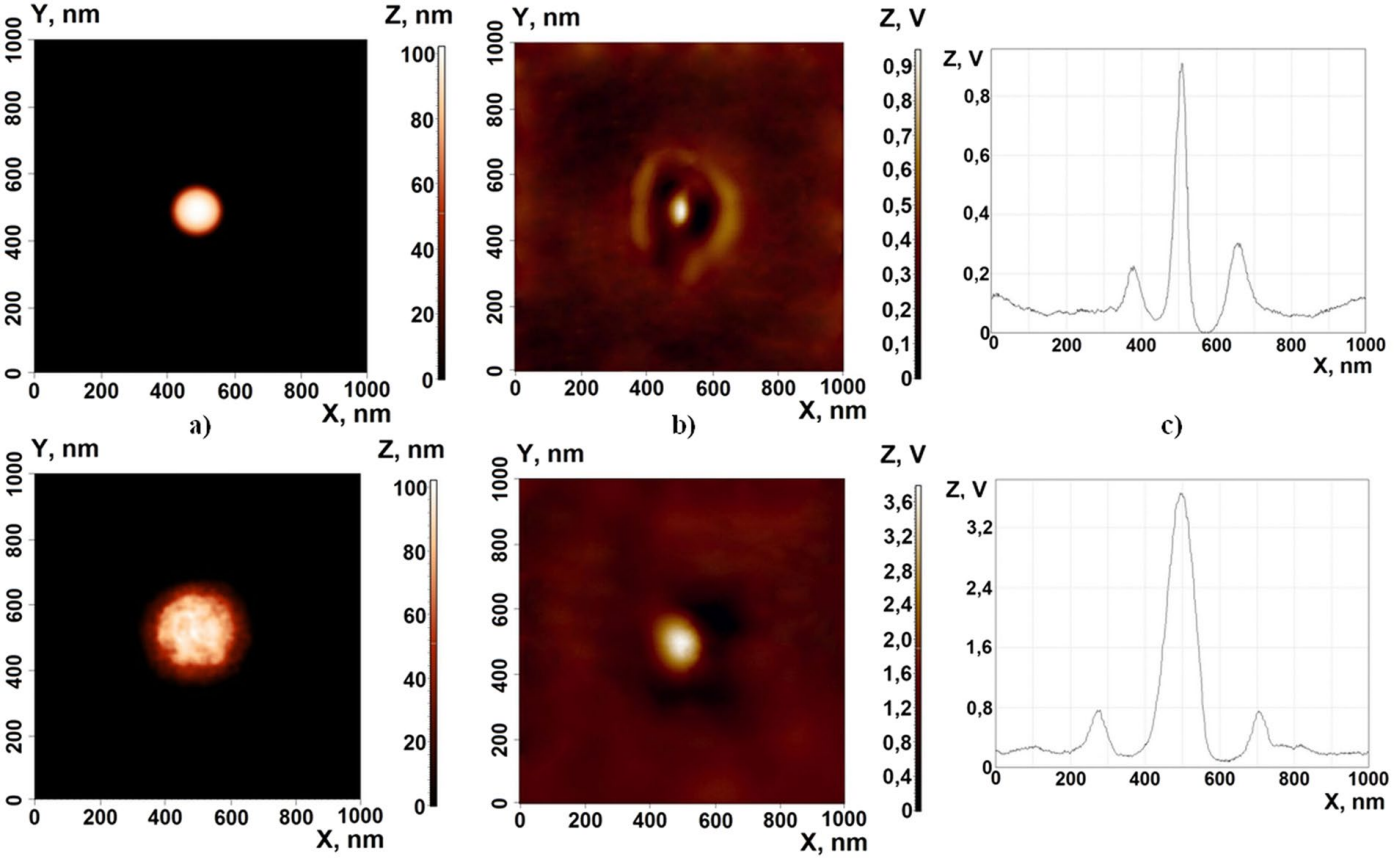
The AFM (**a**) and SNOM images (**b**) of the deposited Si (top panels) and Si-Au (bottom panels) nanoparticles. The panels (**c**) shows the profiles of the corresponding SNOM images (**b**) taken at Y = 500 nm.

Figure 6 compares the AFM and SNOM images of silicon (a) and hybrid gold-silicon (b) nanoparticles. One can see that in both cases the SNOM spectra are characterised by a strong peak at the centre of the particle surrounded by a weaker intensity crown corresponding to the field of the surface localised Mie mode (see also the SNOM profiles at the panels (c)). The hybrid nanoparticles are larger than the pure silicon ones, which is why the diameter of the crown demonstrated by the hybrid particles is approximately twice large than the size of the crown in the SNOM image of pure silicon particles. The most striking difference in the SNOM images of two kinds of nanoparticles is in the amplitude of the central peak that is about 4 times higher in the case of hybrid nanoparticles. The dramatic increase of the near field emission of the hybrid nanoparticles is a signature of the nanoantenna effect of the metallic shell.

The near-field emission focusing is observed in the case of pumping at the wavelength of 510 nm.

## Conclusion

We have experimentally demonstrated that the CW-laser ablation can be used for the synthesis of silicon nanoparticles showing the resonant optical response features in the visible spectral range. Both the pure silicon colloidal NP solutions and the hybrid silicon-gold NP solutions have been studied. The proposed method allows for the efficient synthesis of silicon nanoparticles having an average size of 100 nm. The addition of the gold nanoparticles to the solution and the subsequent laser irradiation modifies the optical response of the system. In particular, it starts featuring broad and intense resonances. In the solutions containing hybrid gold-silicon nanoparticles, the strong electric and magnetic multipole resonances have been observed. The peaks of near-field emission of silicon nanoparticles is increased by a factor of 4 by adding gold. Most likely, this is the manifestation of the nanoantenna effect. These results pave the way to the realization of optical metasurfaces characterized by a controllable variety of functional properties and the strong optical response in the visible spectral range.
